# The *Thoc1* Encoded Ribonucleoprotein Is a Substrate for the NEDD4-1 E3 Ubiquitin Protein Ligase

**DOI:** 10.1371/journal.pone.0057995

**Published:** 2013-02-27

**Authors:** Fei Song, Chuandong Fan, Xinjiang Wang, David W. Goodrich

**Affiliations:** Department of Pharmacology & Therapeutics, Roswell Park Cancer Institute, Buffalo, New York, United States of America; German Cancer Research Center, Germany

## Abstract

Ribonucleoprotein (RNP) complexes form around nascent RNA during transcription to facilitate proper transcriptional elongation, RNA processing, and nuclear export. RNPs are highly heterogeneous, and different types of RNPs tend to package functionally related transcripts. These observations have inspired the hypothesis that RNP mediated mechanisms help specify coordinated gene expression. This hypothesis is supported by the observation that mutations in RNP components can cause defects in specific developmental pathways. How RNP biogenesis itself is regulated, however, is not well understood. The evolutionarily conserved THO RNP complex functions early during transcription to package nascent transcripts and facilitate subsequent RNP biogenesis. THO deficiency compromises transcriptional elongation as well as RNP mediated events like 3′ end formation and nuclear export for some transcripts. Using molecularly manipulated cells and in vitro reconstituted biochemical reactions, we demonstrate that the essential THO protein component encoded by the *Thoc1* gene is poly-ubiquitinated by the NEDD4-1 E3 ubiquitin ligase. Poly-ubiquitinated pThoc1 is degraded by the proteasome. These results indicate THO activity is regulated by the ubiquitin-proteasome pathway, and that this regulation is evolutionarily conserved between yeast and mammals. Manipulation of NEDD4-1 levels has modest effects on *Thoc1* protein levels under steady state conditions, but destabilization of *Thoc1* protein upon treatment with a transcriptional elongation inhibitor is dependent on NEDD4-1. This suggests NEDD4-1 functions in conjunction with other post-translational mechanisms to regulate Thoc1 protein and THO activity.

## Introduction

The co-transcriptional packaging of nascent RNA transcripts into RNP complexes is important for transcriptional elongation, RNA processing, and RNA export from the nucleus [1]. RNP complexes are composed of multiple protein and RNA subunits. They are heterogeneous and dynamic, differing in composition depending on the transcript and the stage within a transcripts life cycle. Potential combinatorial permutations are vast enough to potentially ensure that each transcript is processed by a unique series of RNP complexes [2]. These observations have inspired the hypothesis that co- and post-transcriptional RNP mediated mechanisms support the elaboration of coordinated gene expression programs [3], [4]. Consistent with this hypothesis, mutations in RNP encoding genes can cause specific developmental defects [5]. While the importance of RNP mediated mechanisms for coordinated gene expression is increasingly appreciated, how RNP biogenesis itself is regulated is not well understood.

THO is an evolutionarily conserved RNP complex that accompanies the actively transcribing RNA polymerase II complex [6], [7] and is transferred to nascent RNA transcripts in a RNA cap, splicing, and ATP dependent manner [8], [9]. THO recruits RNA export factors to form larger RNP complexes like TREX [9]–[12]. A deficiency in THO leads to the formation of R-loops, longer than normal stretches of nascent RNA that remain hybridized to the DNA template. R-loops impair transcriptional elongation and increase transcription associated DNA recombination [6], [13]–[16]. Loss of THO also compromises downstream RNP mediated events such as transcript 3′ end formation [17] and nuclear export [18].

THO is a stoichiometric combination of five proteins encoded by the *THOC1*, *THOC2*, *THOC5*, *THOC6*, and *THOC7* genes in metazoan [10]. How THO assembly, stability, and activity are regulated is not well characterized. Proteomic screens have suggested mammalian THO proteins are post-translationally modified by phosphorylation, acetylation, sumoylation, and ubiquitination [19], although these modifications have not been confirmed by targeted studies. In yeast, THO proteins are modified by phosphorylation [20], and the stability of the Hpr1p component appears to be uniquely regulated by the HECT domain containing ubiquitin ligase Rsp5 [21]. Interestingly, THO proteins become unstable in the absence of Hpr1p [22], [23]. These observations suggest Hpr1p is centrally important in regulating yeast THO activity. The mammalian Hpr1p orthologue is encoded by the *Thoc1* gene. Here we test whether the mammalian *Thoc1* encoded protein (pThoc1) is a substrate for the HECT domain containing NEDD4-1 E3 ubiquitin ligase.

## Materials and Methods

### Cell culture and transfection

All cells were cultured in DMEM medium supplemented with 10% FBS (Atlanta Biological Inc.). HeLa and 293 cells were obtained from ATCC. NEDD4-1 knockout murine embryonic fibroblasts have been described previously [24]. HeLa cells engineered to express tetracycline inducible shRNA targeting NEDD4-1 were provided by Xuejun Jiang (Memorial Sloan-Kettering Cancer Center). Plasmid transfection was performed using the FuGENE HD Transfection Regent according to manufacturer's recommendations (Roche). *THOC1* [6] and *NEDD4-1* [25], and His tagged ubiquitin expression plasmids [26] were described previously. Cells were treated with MG132 at 5 µM (Calbiochem) for 24 hours to inhibit proteasome activity. Tetracycline (Sigma) was used at 5 µg/ml to induce *NEDD4-1* targeted shRNA expression. Cycloheximide at 10 µg/ml (Sigma) was used to inhibit protein synthesis. 5,6-Dichlorobenzimidazole 1-β-D-ribofuranoside (DRB) was used at 60 µM (Sigma).

### Protein purification and western blotting

His-Ub expressing cells were lysed in urea buffer A (8 M urea, 0.1 M phosphate, 0.01 M Tris-pH 8.0, 15 mM imidazole, 0.2% Triton X-100), sonicated, and the protein concentration of the cleared lysate quantitated by Bradford assay. His-Ub containing proteins were purified using Ni-NTA agarose beads (Invitrogen) pre-washed in urea buffer A. Bound protein was washed consecutively with urea buffer A, urea buffer B (8 M urea, 0.1 M phosphate, 0.01 M Tris- pH 6.3, 15 mM imidazole, 0.2% Triton X-100), and buffer T1 (25 mM Imidazol, 0.2% Triton X-100, 0.01% SDS). His-Ub containing proteins were eluted by boiling in 2X SDS-PAGE sample buffer containing 200 mM imidazole. Co-immunoprecipitation and western blot analysis using antibodies specific for pThoc1 (GeneTex), NEDD4-1 (Millipore), or actin (Calbiochem) was described previously [6], [27].

### In vitro ubiquitination assay

The reaction was carried out at 30°C for 1 hr in 50 µl reaction buffer (40 mMTris-HCl, pH 7.5, 2 mM DTT, 5 mM MgCl2) containing the following components: 0.5 µg/µl of ubiquitin (Boston Biochem), 50 nM human E1 (Boston Biochem), 400 nM of recombinant UbcH5c, 5 mM ATP, and 25 µl endogenous pThoc1 immunoprecipitated from 1 mg of 293 cell protein lysate. Recombinant His tagged human NEDD4-1 was expressed in SF9 cells and purified with Ni-NTA beads (Novagen). The reaction was terminated by adding 20 µl 4x SDS-PAGE sample buffer and boiling for 10 minutes.

## Results

### Thoc1 is poly-ubiquitinated in intact cells

**Figure 1:**
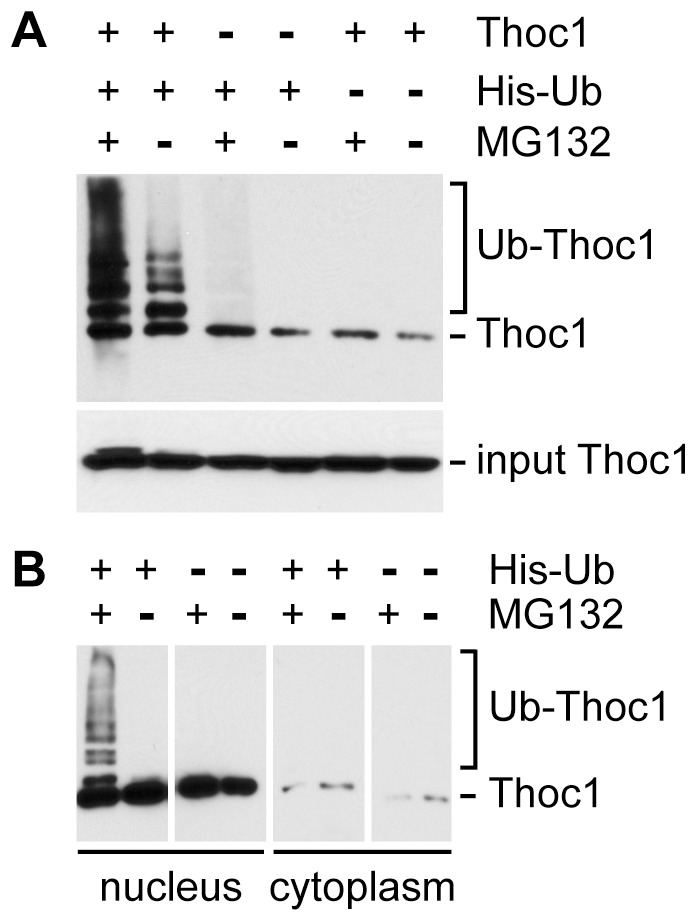
*Thoc1* protein is poly-ubiquitinated in cells. A) HeLa cells were transfected with a *THOC1* expression vector (Thoc1), a vector designed to express histidine affinity tagged ubiquitin (His-Ub), or treated with the proteasome inhibitor MG132. His-Ub containing proteins were purified from total cell protein extracts by metal chelate affinity chromatography, and the purified material analyzed for the presence of pThoc1 by western blotting. Poly-ubiquitinated pThoc1 appears as a ladder of higher apparent molecular weight forms (top panel). Relative input levels of total pThoc1 were also analyzed by western blotting (lower panel). B) HeLa cells were transfected with *THOC1* expression vector (all lanes), the His-Ub expression vector, or treated with MG132 as indicated. His-Ub containing proteins were purified from an equal amount of total nuclear or cytoplasmic protein for each lane, and the presence of pThoc1 detected by western blotting.

We transfected *Thoc1* and histidine tagged ubiquitin (His-Ub) expression vectors into HeLa cells, purified His-Ub containing proteins by metal chelate affinity chromatography, and analyzed the purified proteins for the presence of *THOC1* protein (pThoc1) by western blotting (Figure 1A). Co-purification of multiple, higher molecular weight forms of pThoc1 with His-Ub indicated the exogenously expressed protein was modified by ubiquitin. Accumulation of poly-ubiquitinated pThoc1 increased upon treatment with the proteasome inhibitor MG132, indicating that poly-ubiquitinated pThoc1 was degraded by the proteasome. Endogenous pThoc1 was also poly-ubiquitinated as indicated by the appearance of MG132 sensitive higher molecular weight forms in the absence of transfected *THOC1* expression vector. We note that transfection of the *THOC1* expression vector does not increase total pThoc1 levels significantly over endogenous levels (Figure 1A, input panel), but does significantly increase the levels of poly-ubiquitinated pThoc1. Exogenous pThoc1 is likely in stoichiometric excess over other THO proteins and thus not incorporated into intact THO complexes. Excess, free pThoc1 appears to be preferentially poly-ubiquitinated and degraded by the proteasome.

*THOC1* protein is a predominantly nuclear protein that potentially shuttles to the cytoplasm [28], [29]. We have fractionated transfected and treated HeLa cells into nuclear and cytoplasmic extracts prior to metal chelate affinity chromatography in order to determine where pThoc1 is ubiquitinated. Consistent with published work, most pThoc1 resides within the nucleus, and nuclear pThoc1 is poly-ubiquitinated (Figure 1B). While cytoplasmic poly-ubiquitinated pThoc1 is not detected, the cytoplasmic pThoc1 signal is too low to rule out the possibility that ubiquitinated pThoc1 accumulates in the cytoplasmic compartment.

### NEDD4-1 levels correlate with pThoc1 poly-ubiquitination

**Figure 2:**
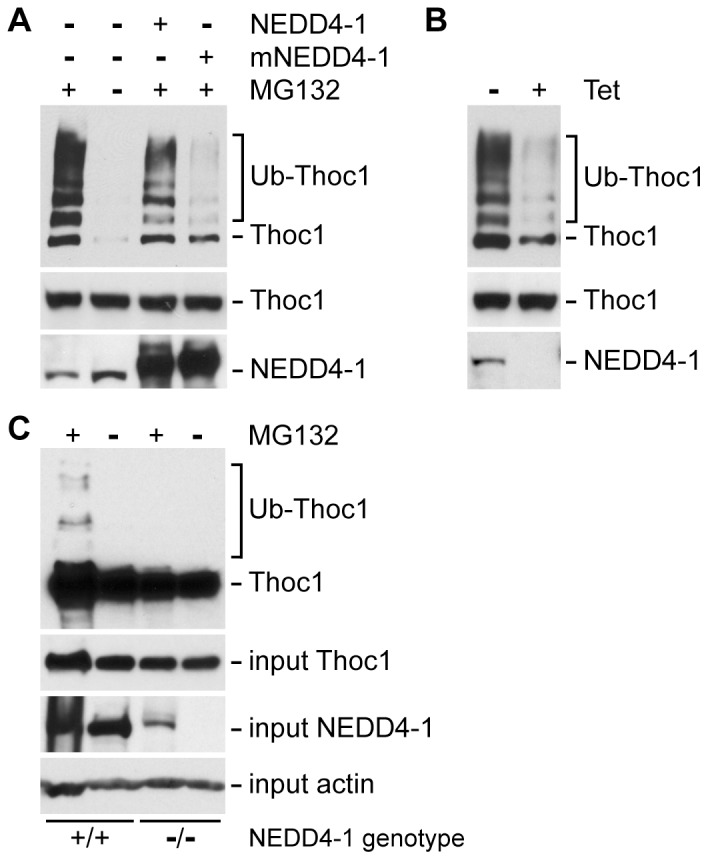
pThoc1 poly-ubiquitination correlates with NEDD4-1 levels in cells. A) HeLa cells were transfected with the *THOC1* and His-Ub expression vectors (all lanes). In addition, cells were transfected with a wild type *NEDD4-1* expression vector (NEDD4-1), a catalytic dead *NEDD4-1* mutant (NEDD4-1 mut), or were treated with MG132 as indicated. Total cell protein extracts were purified by metal chelate chromatography and purified protein analyzed for the presence of pThoc1 by western blotting (top panel). The middle panel shows relative input levels of pThoc1. The bottom panel shows relative input levels of NEDD4-1. B) HeLa cells engineered to express tetracycline inducible *NEDD4-1* targeted shRNA were transfected with *Thoc1* and His-UB expression vectors (both lanes), treated with MG132 (both lanes), or treated with tetracycline. Total cell protein extracts were purified and analyzed as in A). C) Wild type or Nedd4-1 knockout murine embryonic fibroblasts were transfected with the *Thoc1* and His-Ub expression vectors, and treated with MG132 as indicated. His-Ub containing proteins were purified and analyzed for the presence of pThoc1 as above. Input levels of pThoc1 and NEDD4-1 were determined by western blotting while β-actin serves as a loading control. The NEDD4-1 immunoreactive band detected in NEDD4-1 null cells is a non-specific background protein that becomes detectable upon MG132 treatment.

NEDD4-1 is a member of the HECT domain containing E3 ubiquitin ligase family, and we tested whether altering NEDD4-1 levels affected pThoc1 poly-ubiquitination in cells. Transfection of a wild type NEDD4-1 expression vector increases pThoc1 poly-ubiquitination compared to transfection of a catalytically dead NEDD4-1 mutant (Figure 2A). Similarly, silencing NEDD4-1 expression in HeLa cells engineered to express tetracycline inducible, NEDD4-1 targeted shRNA reduces pThoc1 poly-ubiquitination relative to vehicle treated control cells (Figure 2B). pThoc1 poly-ubiquitination is also lower in Nedd4-1 knockout murine embryonic fibroblasts (MEF) than it is in wild type MEFs (Figure 2C). These observations suggest pThoc1 may be a substrate for the NEDD4-1 E3 ubiquitin ligase.

### NEDD4-1 associates with and ubiquitinates pThoc1

**Figure 3:**
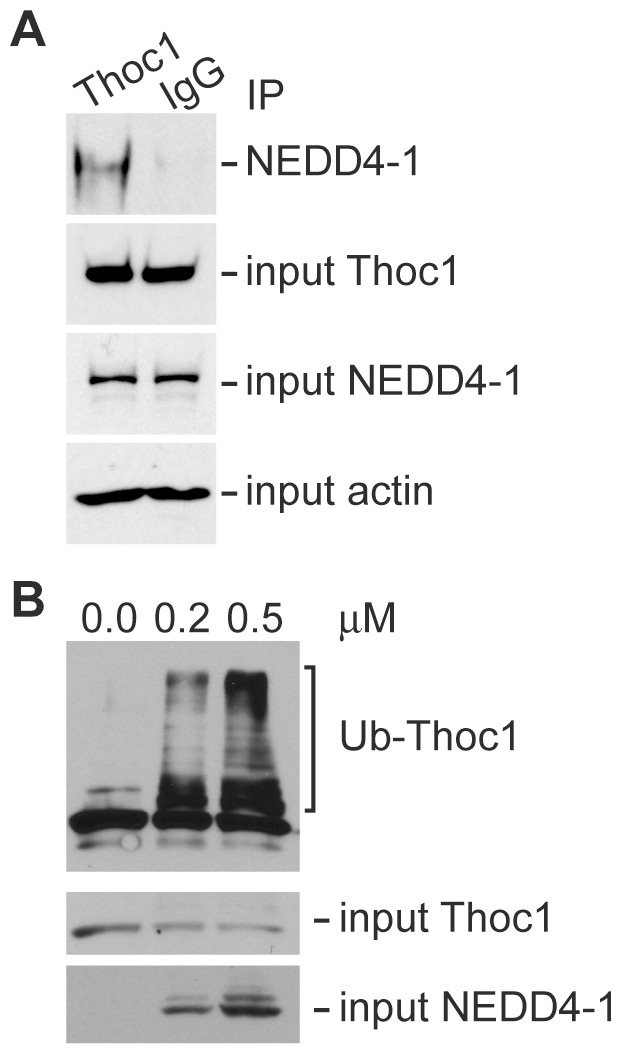
NEDD4-1 binds and ubiquitinates pThoc1. A) 293 cell extracts were immunoprecipitated with a pThoc1 specific antibody or non-specific IgG, and the immunoprecipitates analyzed for the presence of NEDD4-1 by western blotting. Input levels of pThoc1 and NEDD4-1 were assayed by western blotting. β-actin serves as a loading control. B) Immunopurified pThoc1 was mixed with the indicated amount of purified recombinant NEDD4-1 in an in vitro reconstituted ubiquitination reaction. pThoc1 poly-ubiquitination was analyzed by western blotting (top panel). Input levels of pThoc1 or NEDD4-1 (bottom panel) were determined by western blotting.

NEDD4 family E3 ubiquitin ligases are composed of a catalytic C-terminal HECT domain and N-terminal C2 and WW domains that contribute to cellular localization and substrate recognition. pThoc1 contains a LPEY amino acid motif (aa 518–521) that is potentially recognized by NEDD4 family WW domains [30]. To explore the possibility that endogenous pThoc1 and NEDD4-1 physically interact, we analyzed pThoc1 immunoprecipitates for the presence of NEDD4-1 by western blotting. pThoc1 immunoprecipitates do contain detectable levels of co-purifying NEDD4-1, but non-specific IgG immunoprecipitates do not (Figure 3A). We reconstituted NEDD4-1 mediated ubiquitin ligase activity in vitro to test directly whether pThoc1 can serve as a substrate for NEDD4-1. Immunopurified pThoc1 is poly-ubiquitinated by purified recombinant NEDD4-1 in a dose dependent manner in vitro (Figure 3B), and this poly-ubiquitination correlates with a decrease in the remaining non-ubiquitinated pThoc1 as expected (Figure 3B, input panel).

### NEDD4-1 affects pThoc1 stability in cells treated with the transcription elongation inhibitor 5,6-dichloro-1-β-D-ribofuranosylbenzimidazole

**Figure 4:**
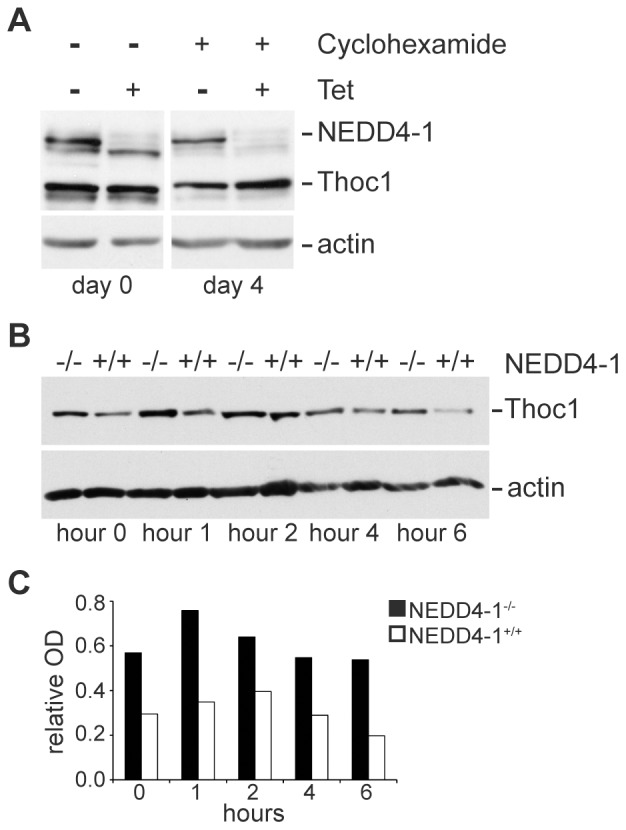
Destabilization of pThoc1 by treatment with DRB is dependent on *NEDD4-1*. A) HeLa cells engineered to express tetracycline inducible shRNA targeting *NEDD4-1* were treated with tetracycline and/or cycloheximide as indicated and cells extracted at the indicated times post cycloheximide treatment. The indicated proteins were analyzed by western blotting. β-actin serves as a loading control. B) Wild type of NEDD4-1 null MEFs were treated with DRB and cycloheximide and extracted at the indicated times post-treatment. *Thoc1* protein levels were assayed by western blotting with β-actin serving as a loading control. C) The western blot in B) was analyzed by densitometry and the optical density of the pThoc1 band relative to the actin band plotted. pThoc1 stability is increased in the absence of *NEDD4-1*.

Under steady state conditions pThoc1 is quite stable [27]. However, poly-ubiquitinated pThoc1 is clearly degraded by the proteasome, suggesting pThoc1 stability is potentially regulated by NEDD4-1 (Figure 1A). To test this, pThoc1 levels have been monitored during cycloheximide treatment in cells with reduced *NEDD4-1* expression levels. Tetracycline inducible *NEDD4-1* gene silencing slows the degradation of existing pThoc1 relative to uninduced cells with normal levels of *NEDD4-1* expression (Figure 4A). Even in the presence of NEDD4-1, however, pThoc1 is quite stable with a half-life on the order of days. The stability of pThoc1 is known to decrease significantly upon treatment with the transcriptional elongation inhibitor 5,6-dichloro-1-β-D-ribofuranosylbenzimidazole (DRB) [6]. Surmising that NEDD4-1 may have a greater impact under conditions that normally result in pThoc1 destabilization, pThoc1 levels were examined over time in wild type or *NEDD4-1* knockout MEFs treated with DRB and cycloheximide. Wild type MEFs show a significant decrease in pThoc1 stability upon treatment with DRB, its protein half-life decreasing to approximately 6 hours (Fig. 4B, C). Relative to wild type MEFs, pThoc1 half-life was significantly longer in *NEDD4-1* knockout MEFs as pThoc1 protein levels do not significantly change during 6 hours of treatment with DRB and cycloheximide. We note that steady state pThoc1 levels are higher in untreated *NEDD4-1* knockout MEFs compared to wild type MEFs (Fig. 4B, C; 0 hour time point).

## Discussion

The results presented here demonstrate that pThoc1, an essential protein component of the mammalian THO RNP complex, is a substrate for the NEDD4-1 E3 ubiquitin ligase. Supporting data includes the detection of pThoc1 poly-ubiquitination, the correlation between NEDD4-1 levels and the extent of pThoc1 poly-ubiquitination, physical association between pThoc1 and NEDD4-1 in cells, and the ability of NEDD4-1 to mediate pThoc1 poly-ubiquitination in an in vitro reconstituted reaction using purified components. NEDD4-1 is a member of the HECT catalytic domain E3 ubiquitin ligase family. The yeast pThoc1 orthologue Hpr1p serves as a substrate for Rsp5p, another member of the HECT domain family of ubiquitin ligases [21]. These observations indicate that the THO RNP complex can be regulated by HECT domain ubiquitin ligases, and that this regulation is evolutionarily conserved from yeast to mammals.

pThoc1 is poly-ubiquitinated by NEDD4-1, poly-ubiquitinated pThoc1 is degraded by the proteasome, and NEDD4-1 status influences pThoc1 levels. These observations demonstrate that pThoc1 stability and thus THO RNP complex activity can be regulated by NEDD4-1. It is seemingly paradoxical that treatment with proteasome inhibitor MG132 has little effect on the steady state levels of endogenous pThoc1 (see Fig. 2). It is also surprising that *THOC1* expression vector transfection does not significantly increase total pThoc1 levels, but does increase the levels of poly-ubiquitinated pThoc1. One possible explanation for these observations is that pThoc1 is very stable when incorporated into an intact THO RNP complex, but is unstable in its free form. Excess free pThoc1 generated by transfection may be particularly susceptible to NEDD4-1 poly-ubiquitination and proteasomal degradation because it cannot be incorporated into stoichiometric, intact THO complexes. Because excess free pThoc1 is unstable, exogenous expression does not significantly alter total pThoc1 levels. This explanation implies that intact THO complexes must be disrupted or post-translationally modified in some way before pThoc1 becomes susceptible to NEDD4-1 mediated poly-ubiquitination. Consistent with this possibility, pThoc1 is destabilized by treatment with the transcriptional elongation inhibitor DRB, and pThoc1 destabilization is dependent on NEDD4-1. This post-translation regulation is expected to facilitate RNP turnover or remodeling during transcription, RNA processing, and export. We note that Rsp5p mediated degradation of Hpr1p is dependent on temperature and ongoing transcription [21], suggesting an analogous situation in yeast.

While NEDD4-1 targets pThoc1 for proteasomal degradation and influences pThoc1 levels under steady state and destabilizing conditions, it is also possible that NEDD4-1 mediated ubiquitination regulates other aspects of pThoc1 function. In addition to regulating protein stability, ubiquitination can alter protein localization, protein interaction, or protein function depending on the type ubiquitin modification [31]. For example, NEDD4-1 may function to alter pThoc1 protein interactions during RNP remodeling and nuclear export. Consistent with this possibility, Rsp5p mediated Hpr1p ubiquitination in yeast facilitates recruitment of the mRNA export receptor Mex67p [32]. Identifying the types of ubiquitin modifications made by NEDD4-1 on pThoc1 and characterizing the effects of these modifications on THO RNP complex formation will be necessary to test this hypothesis. The in vitro reconstituted reaction described here will be useful for such studies.

Dynamic RNP biogenesis is increasingly recognized to contribute to the elaboration of gene expression programs necessary for normal growth and development. How the process of RNP biogenesis itself is regulated is not well understood. The data presented here indicate that the essential THO RNP complex component pThoc1 can be modified by the NEDD4-1 E3 ubiquitin ligase. This observation predicts NEDD4-1 will be an important component in the regulation and coordination of RNP biogenesis mediated by the THO RNP complex. This molecular mechanism likely contributes to the physiological functions attributed to NEDD4-1, and suggests the possibility that post-translational ubiquitination plays a more general role in the regulation and execution of RNP biogenesis and coordinated gene expression.
